# Progress and prospects for accelerating materials science with automated and autonomous workflows

**DOI:** 10.1039/c9sc03766g

**Published:** 2019-09-20

**Authors:** Helge S. Stein, John M. Gregoire

**Affiliations:** a Joint Center for Artificial Photosynthesis , California Institute of Technology , Pasadena , CA 91125 , USA . Email: gregoire@caltech.edu; b Division of Engineering and Applied Science , California Institute of Technology , Pasadena , CA 91125 , USA

## Abstract

Accelerating materials research by integrating automation with artificial intelligence is increasingly recognized as a grand scientific challenge to discover and develop materials for emerging and future technologies. While the solid state materials science community has demonstrated a broad range of high throughput methods and effectively leveraged computational techniques to accelerate individual research tasks, revolutionary acceleration of materials discovery has yet to be fully realized. This perspective review presents a framework and ontology to outline a materials experiment lifecycle and visualize materials discovery workflows, providing a context for mapping the realized levels of automation and the next generation of autonomous loops in terms of scientific and automation complexity. Expanding autonomous loops to encompass larger portions of complex workflows will require integration of a range of experimental techniques as well as automation of expert decisions, including subtle reasoning about data quality, responses to unexpected data, and model design. Recent demonstrations of workflows that integrate multiple techniques and include autonomous loops, combined with emerging advancements in artificial intelligence and high throughput experimentation, signal the imminence of a revolution in materials discovery.

## Abstract 2

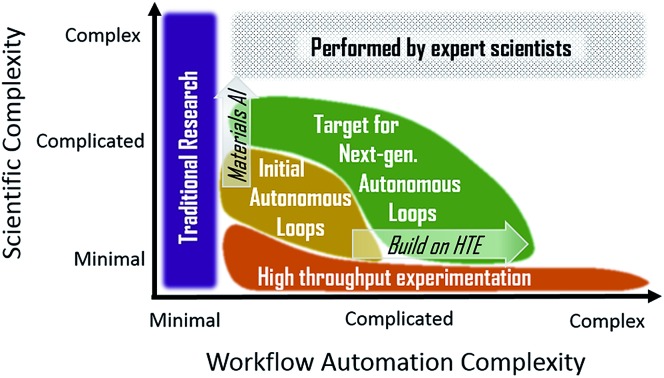
Integrating automation with artificial intelligence will enable scientists to spend more time identifying important problems and communicating critical insights, accelerating discovery and development of materials for emerging and future technologies.

## Introduction

Grand missions, such as combating climate change through proliferation of renewable energy technologies, necessitate technological advancements for which discovery of functional materials is often a prerequisite.^1^,^2^ Historically, transformative materials discoveries have been the result of serendipity from experimenting in a related area and/or decades of systematic materials development.^1^ Early examples of automated synthesis and screening techniques were implemented^3^–^11^ to accelerate both processes,^12^ for example in the identification of a hysteresis-free shape memory alloy.^13^ Continued automation of materials experiments is motivated by potential benefits including lowering per-experiment costs and eliminating human error, and to enable active learning-driven experiments that identify and explore the most promising regions of materials parameter space.^12^,^14^ In solid state materials science, advancements in automation have largely been driven by the combinatorial materials science community, where comprehensive exploration of a high dimensional materials parameter space requires a substantial number of synthesis and screening experiments. While these efforts have provided automation of individual research tasks for a wide variety of materials and functional properties, manual execution of several experiment steps, as well as manual design of experiments and data interpretation, result in partially-automated workflows. The emerging vision of autonomous materials discovery^12^,^15^ requires a higher level of automation. Establishment of an autonomous workflow is referred to as “closing the loop” since complete task-to-task integration is required to allow computer-controlled iteration. Initial^14^,^16^ and ongoing progress towards realizing such closed-loop systems can be tracked by the level of process automation and integration in a workflow.

**Fig. 1:**
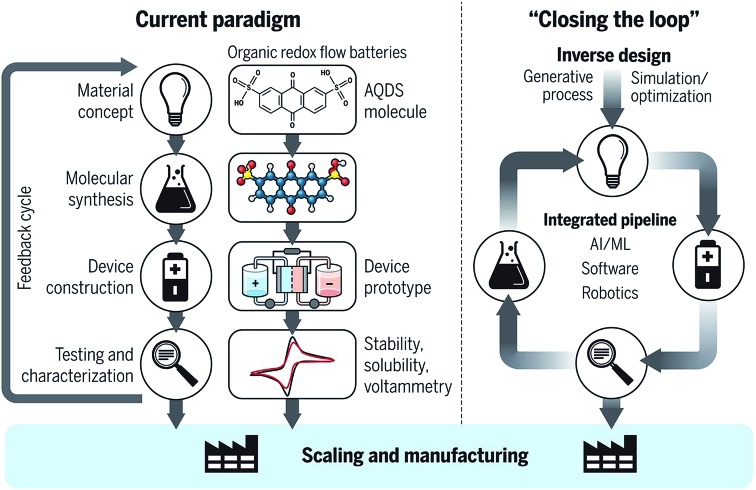
High level comparison of paradigms for materials/molecular sciences. Left: current paradigm exemplified with redox flow batteries. Right: closed-loop discovery utilizing inverse design and a tightly integrated workflow to enable faster identification, scale-up and manufacturing. Figure reproduced from *Science*, **361**, 6400, 360–365 with permission from The American Association for the Advancement of Science.

Sanchez-Lengeling and Aspuru-Guzik^17^ recently described the advent of closed-loop experimentation as a paradigm shift in materials and molecular discovery. The illustration of Fig. 1 provides the high level template of a closed-loop workflow, and in the present work we critically review the progress towards this vision in solid materials experiments. The integration of sequential automated processes is challenging due to the need for mutually compatible parameters and planning, with requirements spanning from a commensurate sample format, to a protocol for decision-making based on results from the prior experiment, and to the identification of measurement failure. To facilitate the analysis of where process integration has been successfully implemented as well as the remaining challenges, we present a framework and ontology for the automation of the materials experiment lifecycle.

The exploration of vast materials spaces (*i.e.* composition, structure, processing, morphology) *via* combinatorial materials science has yielded a wide variety of discoveries and advancements in fundamental knowledge^14^,^18^–^20^ and has additionally produced experiment databases with unprecedented breadth of materials and measured properties, as exemplified by the recent publication of the High Throughput Experimental Materials database (HTEM)^21^ based on photovoltaics materials and the Materials Experiments and Analysis Database (MEAD)^22^ based on solar fuels materials. These compilations of raw and analyzed^23^ data from individual combinatorial materials science laboratories complement the suite of computational materials databases^60^,^61^ as well as a rapidly growing number of materials data repositories including the Citrination platform,^24^ the Materials Data Facility (MDF),^25^ and text mining of the literature.^26^ For the purposes of the present analysis of automating^12^,^16^,^27^ materials science workflows, these databases serve as successful examples of experiment automation and as resources that can be used to accelerate experiment planning, for example by training machine learning models to identify promising materials. In such planning, it is important to note complementary search goals of optimizing a given material property and establishing relationships that represent fundamental materials knowledge. Mapping composition–structure–processing–function relationships^28^–^30^ is a tenet of combinatorial materials research,^28^–^30^ which contrasts with direct implementation of active learning to optimize^31^ one or a few properties without requiring acquisition of data to elucidate the underpinnings of the materials optimization. Indeed the experiment workflow and its operation must be designed to meet the specific research goals, although workflow automation is important for accelerating many different modes of discovery.

We discuss the lifecycle of materials science experiments and the three primary stages of workflow acceleration, (i) the integration of new techniques into traditional research tasks to accelerate process throughput, (ii) the integration of research tasks into a cohesive workflow to mitigate bottlenecks, and (iii) integration of tasks with automated analysis and decisions to close experiment loops and enable autonomous iteration thereof. We find that the solid state materials science community has demonstrated tremendous progress in the first stage, substantial progress in the second stage including high throughput workflows, and seminal demonstrations in the third stage with relatively simple workflows, making concurrent advancement of both the level of autonomy and extent of the workflow a priority research direction.

## The experimental materials science research lifecycle

**Fig. 2:**
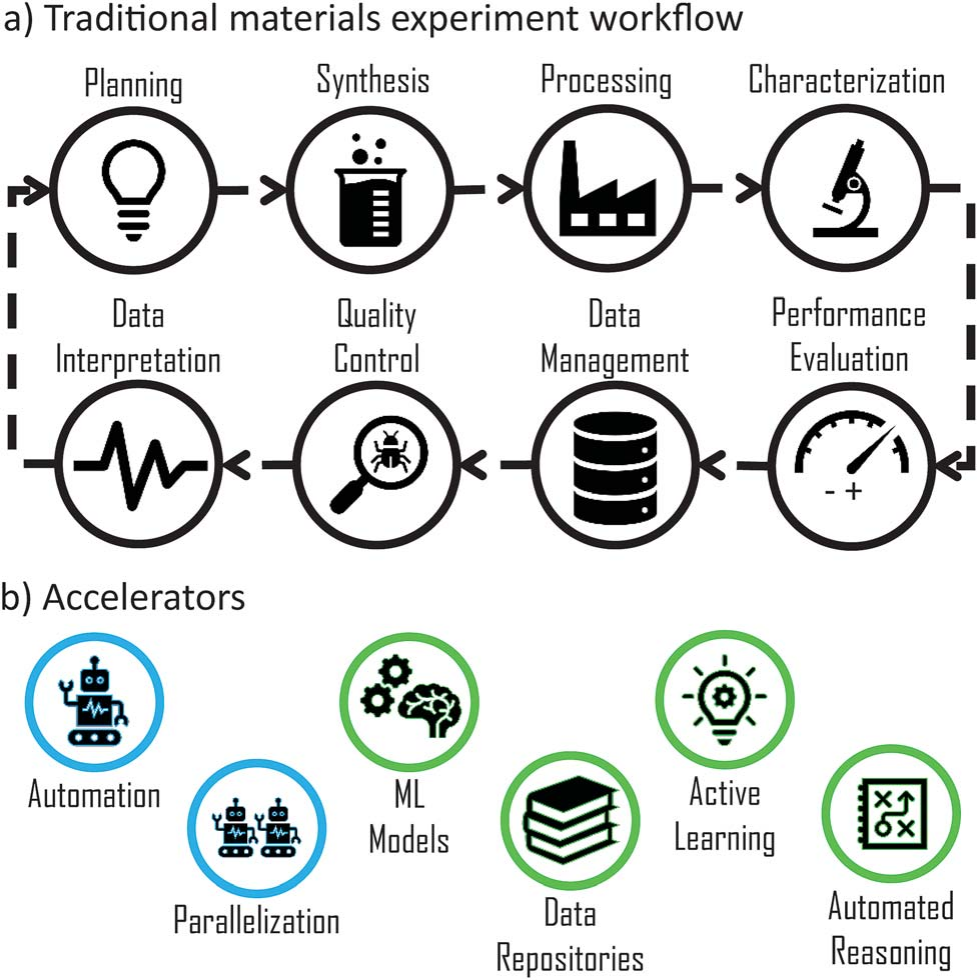
(a) Overview of core research tasks with arrows indicating the cyclic execution of a traditional materials science experimental workflow. (b) Acceleration of each task in a workflow can be obtained by incorporating acceleration technique(s), as represented by these 6 types of accelerators.

At a high level, the experiment lifecycle†
†There are different terms to describe the sequence and interplay of basic research tasks such as materials pipeline, materials highway, or materials platform. for functional materials discovery consists of a set of core research tasks: synthesis, processing, characterization and performance evaluation. This set transcends the specific techniques used to perform each task, and their generality is evident in their consistent discussion in reviews,^1^,^32^ laboratory workflow descriptions,^6^,^33^,^34^ and database designs for high throughput materials science.^5^,^6^,^10^,^32^,^35^–^37^ Often unmentioned, though virtually always performed, are the additional core research tasks of planning, data management, data interpretation, and quality control. Individual and sequences of experiments require these tasks, with the extent and style varying with research strategy. In a traditional materials experiment, the 4 experiment tasks are performed manually, as are the complementary 4 tasks, for example planning *via* a stated hypothesis and data management *via* lab notebooks. The corresponding workflow can be represented as shown in Fig. 2a and represents the foundation on which more advanced and accelerated workflows are built. As noted above, the first stage of workflow acceleration involves implementation of techniques we refer to as “accelerators” into one or more of the workflow tasks. Classifying all possible accelerators is more subjective than the above classification of workflow tasks, and for the present work we find the 6 accelerators noted in Fig. 2b enable effective annotation of experimental workflows from the literature. Some accelerator-task combinations are readily achievable, for example parallelization of processing by annealing multiple materials in a furnace. Other combinations may not be meaningful, such as active learning of data management. Of the many combinations that are both meaningful and impactful, some have been effectively realized while others are opportunities for further experiment acceleration, as summarized below for each accelerator.

### Automation and parallelization

Automated execution of a serial experiment typically involves incorporation of robotics into a traditional experiment. Parallelization typically involves development of custom instrumentation to perform many experiments simultaneously. Both approaches are commonly used in combinatorial materials science where accelerated synthesis techniques include co-sputtering,^6^ co-evaporation,^10^ ink-jet printing,^38^ combinatorial ball-milling,^39^ high-throughput hydrothermal synthesis,^40^,^41^ and bulk ceramic hot-pressing.^42^ Similarly, the acceleration of the characterization of materials properties and evaluation of performance for a target functionality have been the focus of extensive methods development in the past two decades, with notable demonstrations including electrochemical testing,^43^–^46^ X-ray diffraction,^47^–^49^ processing,^9^,^50^,^51^ optical spectroscopy,^52^,^53^ electric properties,^65^,^66^ shape memory,^13^,^54^ and phase dynamics.^9^ These advancements in experiment automation have undoubtedly led to discoveries that would not have been made in the same time frame using traditional techniques. Automation and parallelization-based removal of synthesis and characterization bottlenecks introduces new challenges for further acceleration of materials discovery, which are generally being addressed with data and data science-related accelerators.

### Data repositories

As noted above, the emergence of experiment databases from high throughput experimentation offer opportunities for data-based accelerations. The established uses of data repositories for accelerating research tasks include the data interpretation for crystallography by matching X-ray diffraction patterns to those from a database,^55^ planning synthesis based on phase diagrams,^56^ and planning catalyst performance evaluation using computational databases of Pourbaix stability.^57^,^58^ Data-driven discoveries are typically enabled by a data repository produced *via* careful data management. While guidelines such as FAIR^59^ exist, these general guidelines focus on data dissemination and do not express the data management requirements for establishing autonomous loops, which require fully automated data ingestion and seamless communication between experimental tasks.

### Machine learning

Acceleration by Machine Learning (ML) models encompasses a broad range of applications of computer science algorithms to perform regression, classification or embedding tasks. The recent literature abounds with discussions of the existing and potential impact of ML in materials research. Given recent reviews covering this topic,^62^ the present discussion focuses on its role in experiment workflows. ML-based acceleration of research tasks typically involves either research planning or data interpretation through evaluation of ML models trained on prior data. Representative examples include selection of composition spaces for exploring metallic glasses based on ML predictions of glass forming ability^70^ and identification of ultraincompressible materials.^71^ ML methods have also been developed to accelerate data interpretation in areas including phase mapping from XRD patterns,^18^ microscopy data,^51^ signal identification in spectroscopy data,^73^ annotation of microstructure images,^74^ and visualization of complex compositions.^34^,^73^ ML methods can also be developed into active learning and reasoning techniques, although due to their different roles with respect to experiments, those techniques are discussed separately, as detailed below.

### Active learning

Active learning involves the choice of the next experiment based on an acquisition function that typically requires a prediction for a figure of merit and the uncertainty thereof.^75^ ML models are used for the prediction and uncertainty estimation, with a distinguishing feature of active learning being the need to update the model in real time during execution of the experimental workflow. Active learning is a key component of closed-loop workflows that can ultimately yield self-driving laboratories.^44^ Algorithms such as Phoenics^63^ have been specifically developed for chemistry experiments and integrated into workflow management software such as ChemOS.^64^ The carbon nanotube (CNT) autonomous research system (ARES) project,^65^ which is discussed further below, is an example of a closed-loop system of a workflow where tasks such as data interpretation are readily automated. There have been additional implementations of active learning in materials science to accelerate individual tasks, for example by acquiring only the necessary X-ray diffraction patterns for phase diagram characterization.^66^ Sophisticated examples of active learning in related fields including functional genomics,^67^ separations optimization,^64^ and multi objective molecular optimization for small molecule drug discovery.^68^ While many optimization-oriented searches are amenable to acceleration *via* active learning, its utility for materials discovery has yet to be sufficiently explored and demonstrated, making the above examples a springboard for assessing the ability of active learning to accelerate complex experimental workflows and the generation of fundamental understanding in materials science.

### Automated reasoning

For complex measurement workflows where competing interpretations of the data need to be considered or a model needs to be reinterpreted given the most recent measurements, the data interpretation, quality control, and planning tasks are not readily automated with existing algorithms, motivating the development of automated reasoning to accelerate these tasks with AI methods that mimic and/or supersede human execution of these tasks (*i.e.* “superhuman performance”^69^). Examples of automated incorporation of physics and chemistry-based models into such tasks include tuning the morphology of a thin film based on a structure zone diagram^51^ and fine-tuning the composition to obtain a desired doping type in semiconducting metal oxides based on spinel doping rules.^70^ The opportunity for AI development in this area is the topic of a recent perspective,^69^ and among the promising research directions is the establishment of generative models that expand the purview of active learning to design materials based on desired properties.^71^ While inverse design has been successfully demonstrated for discovery of functional materials,^70^–^73^ integration into automated workflows remains a challenge for solid state materials research. The corresponding high level challenge for closed-loop experimentation of solid state materials is that the scope of a given automated synthesis tool is often quite limited compared to the scope of materials that may be predicted by an active learning or inverse design algorithm. In organic synthesis, for example, there has been more success in developing workflows that encompass the entirety of the synthesis scope of interest, enabling deeper integration of automated reasoning.^17^

## Integration of tasks into a workflow

The most common type of accelerated discovery workflow consists of an automation-accelerated synthesis and an automation-accelerated characterization or performance evaluation, followed by extensive manual analysis, interpretation, and planning of both additional characterization experiments and future iterations of the workflow. Most commonly the highly automated instruments require manual interfacing (*e.g.* alignment, measurement parameter setup, supervision for quality control), where an increased human involvement corresponds to a lower degree of integration. To simplify the present discussion, we consider two classes of task integration with the distinguishing feature being whether expert involvement is required, which designates the integration as “expert mediated” and indicates the integration is incomplete. This level of integration is prone to creating bottlenecks due to the scarcity of experts. Technique integration by robotics is not distinguished from integration by trained technicians in the present work because the resulting impact on workflow throughput requires more in-depth evaluation of the specific workflow.

**Fig. 3:**
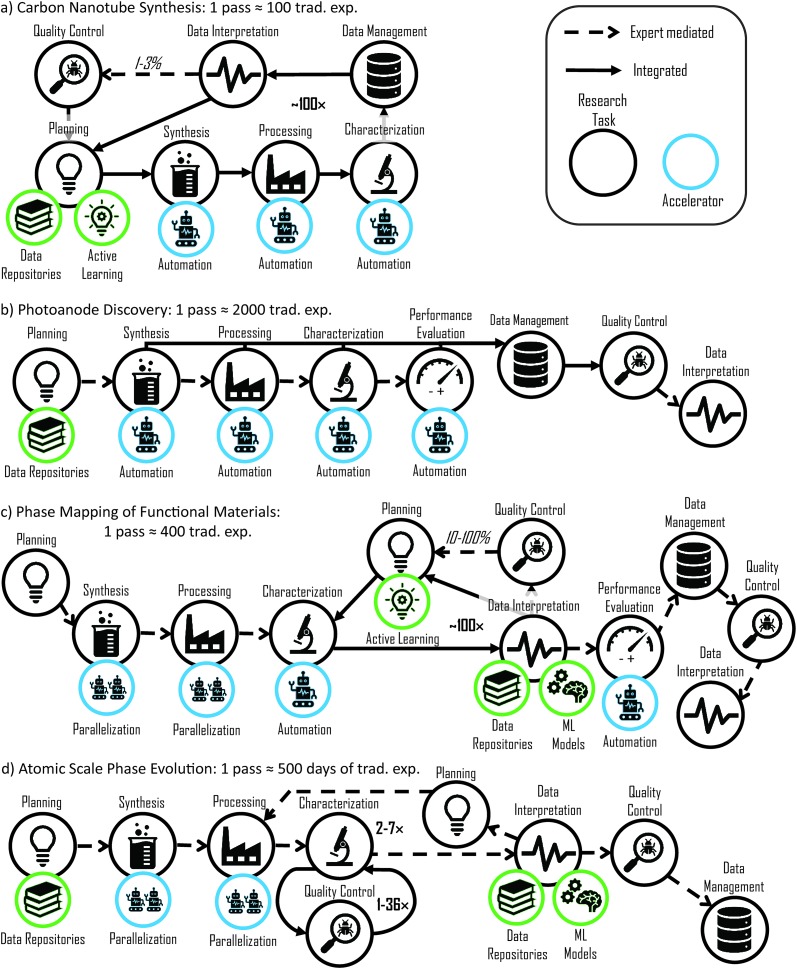
Workflow diagrams of accelerated materials experimentation spanning a range of techniques, strategies and research goals. Based on (a) Nikolaev *et al.*,^14^ (b) Yan *et al.*,^20^ (c) Kusne *et al.*,^66^ and (d) Li *et al.*,^29^ each workflow involves accelerated tasks with various levels of automation and task-to-task integration. The productivity for a single pass through the workflow is noted, corresponding to the number of equivalent traditional experiments for (a)–(c) and duration of traditional experiments for (d). Feedback loops are each labelled with the approximate number of iterations per workflow execution (bold), and in (a) and (c) the percentage of iterations involving expert mediation is also approximated (italics).

To further illustrate how accelerated materials experiments have been integrated, we inspect four reported projects and construct the corresponding workflows in Fig. 3. Each workflow exhibits unique aspects that collectively frame the state of the art in accelerated materials discovery and illustrate the intricacies of workflow acceleration. The scope of each workflow schematic is the sequence of tasks described in the respective publications, and the largest demonstrated equivalent of traditional experimentation is provided for each workflow.

The primary example of closed-loop discovery in solid state materials science is the ARES project for carbon nanotube synthesis. Nikolaev *et al.*^14^ demonstrated optimization of carbon nanotube growth with a workflow that mitigates expert-mediated integration and features acceleration by automation and active learning. Automated control of growth temperature, pressure, and atmospheric conditions enables a unique growth condition in each experiment, with a series of experiments performed by spatially addressing an array of seeds on a substrate. Processing and characterization are intertwined as laser illumination provides both heating and excitation for Raman spectroscopy, producing spectrograms that are analyzed to determine the nanotube growth rate.^14^,^65^ With this materials characterization also providing the figure of merit, the workflow contains no further performance evaluation. The automated data management and interpretation enables closed-loop operation for up to approximately 100 growth experiments planned by active learning-based selection of growth conditions. Expert intervention in this closed loop occurs occasionally (estimated to be 1–3%) to assess the quality of the active learning and adjust the objective as necessary. Upon exhaustion of the array of CNT growth seeds, manual intervention is required to change samples and restart the workflow.

The photoanode discovery pipeline in Fig. 2b represents the tiered screening by Yan *et al.*^20^ that includes both theory and experiment-based down-selection of candidate metal oxides. With respect to the experiments, the computational screening is an accelerant and represented as such in the planning task. The Materials Project database^60^ serves as the primary repository, with additional calculations specific to photoanode screening, and while these calculations are critical to the success of the work, they are not fully integrated into the experimental workflow. Synthesis, processing, characterization, and performance evaluation are accelerated using automation, with tens to thousands of materials being synthesized or measured automatically. While this sequence of tasks is in principle amenable to more autonomous operation, setup and selection on meaningful experimental conditions are chosen by an expert, resulting in expert mediated linkages in the workflow. The heavy use of parallelization and automation is supported by automatic data management and quality control, with data interpretation requiring expert mediation. A key attribute of this workflow is the establishment of automated techniques for a large breadth of experimental tasks, from synthesis to performance evaluation, that can operate on libraries with up to *ca.* 2000 unique materials.^74^ The research strategy involves collection of combinatorial materials datasets that facilitate data interpretation and scientific discovery, as well as evaluation of every prediction from the computational screening to assess its efficacy. These aspects of the research limit the value of further task-to-task integration and application of active learning, with the broader message being that the impact of the closed-loop concept varies with research strategy and goals.

The workflow of Fig. 3c describes a different implementation of combinatorial materials science for studying functional materials where synthesis, processing and performance evaluation are accelerated by parallelization and automation with expert-mediated integration similar to that of Fig. 3b. The unique aspect of this work is the use of an active learning loop in the middle of the workflow to accelerate the mapping of phase boundaries in a composition library, demonstrating the use of active learning in a sub-workflow to accelerate a bottleneck experiment (and save valuable beamline time). The synchrotron X-ray diffraction (XRD) characterization described by Kusne *et al.*^66^ includes on-the-fly data interpretation and automated selection of the next composition for XRD measurements, with occasional expert supervision of the clustering-based identification of pure-phase patterns.

The atomic-scale phase evolution workflow by Li *et al.*^29^ illustrated in Fig. 3d uses a specialized nanometer sized reactor to assess phase stability with *ca.* 1 hour of experiment time yielding the same data as over 500 days of annealing in traditional bulk experiments. Using data repositories of phase diagrams and stability ranges of multicomponent complex metal alloys to plan synthesis, an array of 36 reactors is deposited, for example with equiatomic mixtures of the Cantor alloy Cr–Mn–Fe–Co–Ni.^75^ The loop in this workflow is based on the step-wise annealing of the reactor array with subsequent atom probe tomography (APT) characterization after each processing step. Each APT characterization involves destruction of one of the reactors, and the number of reactors is made to be several times larger than the number of processing steps due to routine failure of the APT measurement. The critical advancement enabled by a small autonomous loop is the real-time monitoring of APT data acquisition with well-integrated quality control. Data interpretation is performed by comparison to external data and visualization is done through a machine learning model.^30^,^76^ The richness of the APT data coupled with significant annealing time reduction yields high throughput knowledge generation even though the workflow contains mostly expert-mediated integration of tasks. Increased autonomy in the workflow would only be warranted after substantial advances in automated data interpretation.

For each of these workflows, the nominal time to execute the entire workflow is on the order of 1 day. The equivalent number of passes through a traditional workflow, or the number of days of traditional experimentation to produce the equivalent data, provides the nominal acceleration factor of the workflow, which is only equal to the acceleration factor of knowledge discovery if the selection of experiments and quality of the resulting data is equivalent to those of traditional experiments. Assessment of such data value is beyond the scope of the present discussion but remains a critical consideration for quantifying workflow acceleration, particularly in settings where the research goals involve understanding the underlying materials science as opposed to performance optimization.

## Conclusions and outlook

The urgent need for better materials demands faster turnaround cycles from basic research, such that better, more efficient, more eco friendly, and more economically viable materials can enter the market sooner than the traditionally observed 40 years.^1^ Accelerated materials experiment workflows have been demonstrated to increase throughput by up to a few orders of magnitude compared to traditional methods. Surveying the reported workflows reveals two primary areas for workflow sophistication, the integration of sequential tasks without requiring expert involvement and the expansion of feedback loops to incorporate a larger fraction of the workflow tasks. The ARES workflow achieves both of these goals with a relatively small workflow compared to the functional materials discovery research where the variety of characterization and performance evaluation experiments increases the number of workflow tasks as well as the demands on data management, data interpretation, and quality control.

**Fig. 4:**
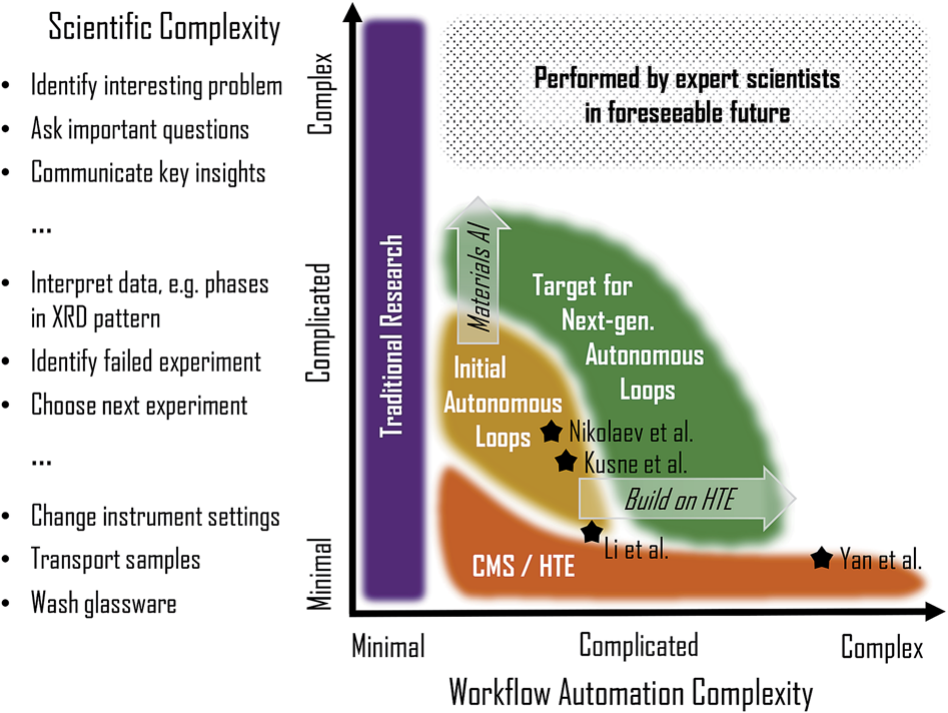
Visualization of the landscape of materials experiment workflow in terms of the scientific complexity of automated tasks and the workflow automation complexity, which is based on the number, variety, speed, and difficulty of experimental steps in the workflow. The advancements in combinatorial materials science and high throughput experimentation (CMS/HTE) have been largely along this latter (horizontal) axis, and initial demonstrations of autonomous loops have made progress on the former (vertical) axis with automation of more intellectually challenging research tasks. The nominal location of the 4 workflows from Fig. 3 are noted by stars. While research will push the frontier of automated experiments along both axes (arrows with italics), the most complex scientific tasks will remain the responsibility of human experts for the foreseeable future.

To visualize progress to date and the expected advances from ongoing research, Fig. 4 illustrates the continuum of materials workflows in terms of the scientific complexity and workflow automation complexity. To elucidate our intended meaning of scientific complexity, representative tasks spanning minimal complexity to very complex are listed. Arguably the most important aspect of a successful science program is the ability to identify interesting problems and ask the important questions that guide research activities. These tasks are beyond the purview of present autonomous research and will be for the foreseeable future. Advances in natural language processing for materials science may automate aspects of scientific communication, but critical analysis of the literature and communication of the insights provided by a given experiment will continue to rely on human intellect for the foreseeable future.

Determining the most effective advancements in a materials experiment workflow requires critical evaluation of bottlenecks for progress against the research goals. Even when expert mediation is required between tasks, workflow throughput is often limited by the manual steps at the front and back ends of automated experiments. These peripheral activities, which fall under the intermediate “complicated” level of scientific complexity in Fig. 4, can be difficult (or currently impossible) to fully automate due to the routine use of expert knowledge, for example in judgement of data quality based on extensive previous experience with related data. Advances in artificial intelligence (AI) for materials encompasses a wide variety of strategies for addressing these challenges, which will be critical for expanding the scope of autonomous loops. This approach to pushing the frontier of materials workflows is illustrated by the “Materials AI” arrow in Fig. 4 and will ideally accompany the expansion of autonomous loops to include more complex and a larger variety of experimental tasks. This complementary approach to pushing the frontier of materials workflows is illustrated by the “Build on HTE” arrow due to the demonstrated successes in experiment automation from the high throughput experimentation community. The ability to leverage this existing work makes autonomous workflows more readily extendable into complex automation as compared to the extremes of complex scientific reasoning.

An outstanding question with regard to the next generation of experimental workflows is how to best combat human biases that can severely limit innovation.^77^ Advanced autonomous experimentation may remove biases within a given search space through computationally designed experiments. However, the scope of the search space is limited by both instrument capabilities and active learning strategy, whose designs originate with human identification of the materials space of interest. To the extent that human biases disseminate from the “complex” scientific tasks of Fig. 4, bias removal within an autonomous workflow must be complemented by sociological solutions for removing bias in decisions beyond the experiment workflow.

We are aware of several research groups that are building autonomous experiments in the "next generation" regime of Fig. 4, including emerging reports from perovskite synthesis^78^ and molecular materials for of organic photovoltaics^79^ and organic hole transport materials.^80^ Continuation of these concerted efforts to increase automation and develop tailored AI algorithms will enable the materials science community to realize a paradigm shift in scientific discovery where expert scientists can dedicate a substantially larger fraction of their time to performing the critical tasks of identifying important problems and communicating critical insights.

## Conflicts of interest

There are no conflicts to declare.

## References

[cit1] Alberi K. (2019). J. Phys. D: Appl. Phys..

[cit2] Report of the Clean Energy Materials Innovation Challenge Expert Workshop January 2018, Mission Innovation, 2018, http://mission-innovation.net/wp-content/uploads/2018/01/Mission-Innovation-IC6-Report-Materials-Acceleration-Platform-Jan-2018.pdf.

[cit3] Xiang X.-D. and Takeuchi I., Combinatorial Materials Synthesis, CRC Press, 2003.

[cit4] Koinuma H., Takeuchi I. (2004). Nat. Mater..

[cit5] Maier W. F., Stöwe K., Sieg S. (2007). Angew. Chem., Int. Ed..

[cit6] Ludwig A., Zarnetta R., Hamann S. (2008). J. Mater. Chem. A.

[cit7] Long C. J., Bunker D., Li X., Karen V. L., Takeuchi I. (2009). Rev. Sci. Instrum..

[cit8] Keller D. A. (2015). ACS Comb. Sci..

[cit9] Li Z., Ludwig A., Savan A., Springer H., Raabe D. (2018). J. Mater. Res..

[cit10] Cawse J. N. (2001). Acc. Chem. Res..

[cit11] Chan E. M. (2015). Chem. Soc. Rev..

[cit12] Saikin S. K., Kreisbeck C., Sheberla D., Becker J. S., Aspuru-Guzik A. (2019). Expert Opin. Drug Discovery.

[cit13] Cui J. (2006). Nat. Mater..

[cit14] Nikolaev P. (2016). npj Comput. Mater..

[cit15] Tabor D. P. (2018). Nat. Rev. Mater..

[cit16] Dimitrov T., Kreisbeck C., Becker J. S., Aspuru-Guzik A., Saikin S. K. (2019). ACS Appl. Mater. Interfaces.

[cit17] Sanchez-Lengeling B., Aspuru-Guzik A. (2018). Science.

[cit18] Umehara M. (2019). npj Comput. Mater..

[cit19] Ludwig A. (2019). npj Comput. Mater..

[cit20] Yan Q. (2017). Proc. Natl. Acad. Sci. U. S. A..

[cit21] Zakutayev A. (2018). Sci. Data.

[cit22] Soedamadji E., Stein H., Suram S., Guevarra D., Gregoire J. (2019). npj Comput. Mater..

[cit23] Gomes C. P., MRS Commun. , 1 –9 10.1557/mrc.2019.50 , , undefined/ed .

[cit24] O'Mara J., Meredig B., Michel K. (2016). JOM.

[cit25] Blaiszik B. (2016). JOM.

[cit26] Weston L. (2019). J. Chem. Inf. Model..

[cit27] Häse F., Roch L. M., Aspuru-Guzik A. (2019). Trends in Chemistry.

[cit28] Suram S. K. (2016). ACS Comb. Sci..

[cit29] Li Y. J., Savan A., Kostka A., Stein H. S., Ludwig A. (2018). Mater. Horiz..

[cit30] Stein H. (2019). Mater. Horiz..

[cit31] Suram S. K., Pesenson M. Z. and Gregoire J. M., High Throughput Combinatorial Experimentation + Informatics = Combinatorial Science, in Information Science for Materials Discovery and Design, ed. T. Lookman, F. J. Alexander and K. Rajan, Springer International Publishing, 2016, pp. 271–300, 10.1007/978-3-319-23871-5_14.

[cit32] Potyrailo R. A., Mirsky V. M. (2008). Chem. Rev..

[cit33] Gregoire J. M., Xiang C., Liu X., Marcin M., Jin J. (2013). Rev. Sci. Instrum..

[cit34] Sliozberg K. (2015). ChemSusChem.

[cit35] Curtarolo S. (2013). Nat. Mater..

[cit36] Zakutayev A., et al., High Throughput Experimental Materials Database, 2017, 10.7799/1407128.

[cit37] Maier W. F. (2019). ACS Comb. Sci..

[cit38] Liu X. (2012). Nano Lett..

[cit39] Li B. (2012). ACS Comb. Sci..

[cit40] Weng X. (2009). J. Comb. Chem..

[cit41] Jin R., Chen G., Pei J., Yan C. (2012). New J. Chem..

[cit42] Stegk T. A., Janssen R., Schneider G. A. (2008). J. Comb. Chem..

[cit43] Jin J., Gregoire J. M. and Xiang C., Scanning Drop Sensor, 2013, pp. 1–12.

[cit44] Mardare A. I., Ludwig A., Savan A., Hassel A. W. (2013). Electrochim. Acta.

[cit45] Grote J. P., Zeradjanin A. R., Cherevko S., Mayrhofer K. J. J. (2014). Rev. Sci. Instrum..

[cit46] Schuppert A. K., Topalov A. A., Katsounaros I., Klemm S. O., Mayrhofer K. J. J. (2012). J. Electrochem. Soc..

[cit47] Takeuchi I., Long C. J., Famodu O. O. (2005). Rev. Sci. Instrum..

[cit48] Long C. J., Bunker D., Li X., Karen V. L., Takeuchi I. (2009). Rev. Sci. Instrum..

[cit49] Gregoire J. M. (2014). J. Synchrotron Radiat..

[cit50] Bell R. T. (2016). ACS Comb. Sci..

[cit51] Stein H. (2015). Phys. Status Solidi A.

[cit52] Schwarting M., Siol S., Talley K., Zakutayev A., Phillips C. (2017). Materials Discovery.

[cit53] Mitrovic S. (2015). Rev. Sci. Instrum..

[cit54] Zarnetta R. (2010). Adv. Funct. Mater..

[cit55] International Centre for Diffraction Data, ICDD, Powder Diffraction File. Powder Diffraction File, Newtown Square, Pennsylvania, USA.

[cit56] Baker H., ASM handbook, ASM international, 1992, vol. 3.

[cit57] Persson K. A., Waldwick B., Lazic P., Ceder G. (2012). Phys. Rev. B: Condens. Matter Mater. Phys..

[cit58] Singh A. K. (2017). Chem. Mater..

[cit59] Wilkinson M. D. (2016). Sci. Data.

[cit60] Jain A. (2013). APL Mater..

[cit61] Curtarolo S. (2012). Comput. Mater. Sci..

[cit62] Correa-Baena J.-P. (2018). Joule.

[cit63] Häse F., Roch L. M., Kreisbeck C., Aspuru-Guzik A. (2018). ACS Cent. Sci..

[cit64] Roch L. M., et al., ChemOS: An Orchestration Software to Democratize Autonomous Discovery, 2018, 10.26434/chemrxiv.5953606.v1. PMC7161969 32298284

[cit65] Nikolaev P., Hooper D., Perea-López N., Terrones M., Maruyama B. (2014). ACS Nano.

[cit66] Kusne A. G. (2014). Sci. Rep..

[cit67] King R. D. (2009). Science.

[cit68] Gómez-Bombarelli R. (2018). ACS Cent. Sci..

[cit69] Gomes C. P., Selman B., Gregoire J. M. (2019). MRS Bull..

[cit70] Paudel T. R., Zakutayev A., Lany S., d'Avezac M., Zunger A. (2011). Adv. Funct. Mater..

[cit71] Perkins J. D. (2011). Phys. Rev. B: Condens. Matter Mater. Phys..

[cit72] Zakutayev A. (2016). J. Mater. Chem. A.

[cit73] Yu L., Kokenyesi R. S., Keszler D. A. and Zunger A., Inverse Design of High Absorption Thin-Film Photovoltaic Materials. Advanced Energy Materials, 2013, available at: https://onlinelibrary.wiley.com/doi/abs/10.1002/aenm.201200538, accessed: 18th July 2019.

[cit74] Newhouse P. F. (2017). Chem. Mater..

[cit75] Cantor B., Chang I. T. H., Knight P., Vincent A. J. B. (2004). J. Mater. Sci. Eng. A.

[cit76] Kruskal J. B. (1964). Psychometrika.

[cit77] Jia X. (2019). Nature.

[cit78] Pendleton I. M., Cattabriga G., Li Z., Najeeb M. A., Friedler S. A., Norquist A. J., Chan E. M., Schrier J. (2019). MRS Commun..

[cit79] Langner S., et al., Beyond Ternary OPV: High-Throughput Experimentation and Self-Driving Laboratories Optimize Multi-Component Systems, arXiv:1909.03511 [physics], 2019. 10.1002/adma.201907801 32049386

[cit80] MacLeod B. P., et al., Self-driving laboratory for accelerated discovery of thin-film materials, arXiv:1906.05398 [cond-mat, physics:physics], 2019. 10.1126/sciadv.aaz8867 PMC7220369 32426501

